# The Structure of Storage Triacylglycerols of Mature Seeds of *Lunaria rediviva* L., a Hyperaccumulator of Very Long-Chain Monounsaturated Fatty Acids, from the Perspective of Statistical Distribution Theories and New Insights Based on Simple Calculations

**DOI:** 10.3390/plants14040612

**Published:** 2025-02-18

**Authors:** Roman Sidorov, Giorgi Kazakov, Vasily Kotsuba, Tatiana Tyurina

**Affiliations:** 1 K. A. Timiryazev Institute of Plant Physiology, Russian Academy of Sciences, Botanicheskaya Street 35, Moscow 127276, Russia; kazakov.giorgi.vladimirovich@yandex.com (G.K.); tyurina.tatiana812@gmail.com (T.T.); 2 Federal Research Center “Fundamentals of Biotechnology”, Russian Academy of Sciences, Leninsky Prospect, 33, Build. 2, Moscow 119334, Russia; vasilyk@yandex.ru; 3 Institute of Comprehensive Exploitation of Mineral Resources, Russian Academy of Sciences, Kryukovsky Cul-de-Sac, Moscow 111020, Russia

**Keywords:** *Lunaria rediviva*, mature seeds, storage oils, triacylglycerols, very long-chain unsaturated fatty acids, Vander Wal model, RP-HPLC-PDA/ESI-MS, positional-species composition

## Abstract

This article represents the first consideration of the peculiarities of the fatty acid (FAs) composition and structure of storage triacylglycerols (TAGs) of the relict plant *Lunaria rediviva* L. The composition of storage TAGs was found to comprise 21 individual FAs, with an unsaturated FA content of 96.8%. Additionally, monounsaturated acids with a very long chain (VLCFAs), specifically C20:1–C24:1, constituted over 60% of the total FAs. The ethylene bond position isomers of unsaturated FAs were accurately identified and the presence of unusual isomers, including 20:1Δ13, 22:1Δ15, and 24:1Δ17 acids. Furthermore, the unusual minor 24:2Δ15,18 acid was identified and characterised for the first time. The pathways of the mentioned VLCFA’s biosynthesis have been proposed. The distribution of FA acyls between the *sn* positions of triacylglycerols was found to be highly specific. Thus, VLCFAs exclusively acylate the α positions of the carbon atoms of the glycerol residue of the TAG molecule (*sn*-1 and *sn*-3 positions), while unsaturated C18 acids exclusively acylate the β-carbon atom (*sn*-2 position). The composition of the molecular species of TAGs was analysed using a calculation method based on the Vander Wal model and by RP-HPLC-ESI-MS. A significant discrepancy from the statistical model was observed, indicating a preference for the formation of symmetrical TAGs, such as *sn*-1,3-dierucoyl-2-oleoyl-glycerol and related molecular species. This observation led to the formulation of a hypothesis regarding the potential existence of at least two specialised enzyme isoforms involved in the biosynthesis of such TAGs via the Kennedy pathway, exhibiting unusual substrate specificity. Consequently, this plant can be regarded not only as a producer of unusual molecular types of triacylglycerols but also as a source of genetic material for the search of genes encoding the aforementioned enzymes with unusual substrate specificity.

## 1. Introduction

The genus of plants *Lunaria* Tourn. ex L., 1753, from the Brassicaceae family comprises a small number of rare endangered species of annual and perennial plants related to the relics of the Tertiary period [1]. In numerous countries, plants belonging to this genus are under protection, given that the area of certain species are recognised as declining. The genus comprises three species: the perennial *Lunaria rediviva* L., the annual *Lunaria annua* L., and the endemic species *Lunaria telekiana* Javorka, which grows exclusively in Albania, Montenegro, and Kosovo [2,3].

The distribution of plants in this genus is considered to be cosmopolitan, meaning that they are present across Europe, extending from the northern to the southern regions, as well as from the western to the eastern parts of the continent. Notable examples of successful introduction and cultivation include species such as *L. annua* and *L. rediviva*, which have been cultivated in Japan, Pakistan, Tasmania, Ireland, Norway, Canada, and several US states, including Illinois, Ohio, Pennsylvania, and Massachusetts [4].

*Lunaria rediviva* L., which is more commonly referred to as perennial honesty, is a perennial herb distinguished by its hairy stems. This plant is capable of growing to a height of 1 m (3.3 ft) and possesses large, pointed oval leaves with marked serrations. It produces clusters of fragrant, pale pink flowers (Figure S1) in the springtime, which are subsequently followed by translucent oval silicles.

A number of outdated manuals on herbal medicine published in the USSR mention that a decoction of perennial honesty seeds was used as part of a complex therapy for epilepsy, and a diuretic effect of the decoction was also noted [5]. To date, the phytochemical compositions of various organs of this plant remain unstudied.

Conversely, other species within the genus have been the subject of more extensive research, particularly with regard to the composition of the fatty oil present in the seeds of *Lunaria annua* L. It accounts for up to 37% of the dry weight [6].

It has long been established that the oil extracted from mature seeds of this species is rich in very long-chain monounsaturated fatty acids, including gondoic (20:1Δ11), erucic (22:1Δ13), and nervonic (24:1Δ15) [6] acids. Previously, these fatty acids were primarily utilised as raw materials in the synthesis of industrial biodegradable lubricants [7], anionic surfactants [8,9], molecular probes for detecting viscosity fluctuations in media [10], and as biofuels with specific properties [11].

Recent studies have demonstrated the significant nutraceutical value of the aforementioned fatty acids, along with their therapeutic potential. For instance, gondoic acid has been found to possess antibacterial properties [12]. Furthermore, recent research has revealed that gondoic acid effectively suppresses the progression of inflammation in key liver macrophages (Kupffer cells) [13]. In addition, levels of gondoic and nervonic acids in the total lipids of human erythrocytes have been shown to be a reliable and independent predictor of mortality from cardiovascular diseases [14].

Erucic acid has been recognised as a causative agent of hepatic steatosis for a considerable duration [15]. This can be attributed to the fact that erucic acid stimulates the accumulation of malonyl-CoA in hepatocytes during peroxisomal β-oxidation. Consequently, this results in the inhibition of β-oxidation of other fatty acids [15]. Furthermore, it has been demonstrated that erucic acid can trigger the accumulation of triacylglycerols in myocardial tissue, resulting in lipidosis, which, in turn, has been shown to reduce cardiac contractility [16,17]. Consequently, this has prompted some researchers to categorise erucic acid as a natural toxin [18]. These considerations have encouraged breeders to concentrate on cultivating erucic acid-free oilseed varieties, including canola and rapeseed.

Recent studies have identified the potential benefits of erucic acid, with findings demonstrating its antimalarial properties against chloroquine-resistant strains of *P. falciparum*. In vivo studies revealed that methyl erucate increased the survival rate and lifespan of infected mice [19]. In addition, a notable study disclosed the remarkable potency of erucic acid in counteracting the influenza A virus, as evidenced by its ability to impede the production of proinflammatory mediators and interferons induced by viral RNA [20]. Furthermore, recent research in mouse models has demonstrated that the administration of erucic acid at a dosage of 3 mg per kg of body weight enhances memory function in healthy mice and retards the progression of memory impairment induced by scopolamine [19]. Consequently, the authors of the study proposed that erucic acid could be considered as a potential new therapeutic agent for the treatment of diseases associated with cognitive decline, such as Alzheimer’s disease [21].

Nervonic acid was first identified in substantial quantities in the sphingolipids of the white matter in the human brain, thus earning its name. It also plays a role in the formation of myelin fibers [22,23]. Its name thus indicates the important role of this acid in neurophysiological and pathophysiological processes. Recent studies have demonstrated a dosage of 70 mg per kg of body weight to reduce symptoms of depressive behaviour in mice subjected to high levels of stress and to reduce or completely compensate for stress-induced demyelination of the medial prefrontal cortex tissues [24]. A similar outcome was obtained by another group of researchers who studied the role of nervonic acid in the restoration of the brain and cognitive functions in rats after a stroke [25]. The anti-inflammatory effects of nervonic acid have also been successfully proved in studies of Alzheimer’s disease [26].

In 2023, it was demonstrated in mouse models of acute enterocolitis that nervonic acid contributes to the restoration of intestinal barrier function by reducing the secretion of pro-inflammatory factors. This outcome was attributed primarily to the ability of nervonic acid to regulate the citrate cycle, amino acid metabolism, pyrimidine, and purine metabolism in mice, thereby protecting them from colitis [27]. Consequently, the authors of the study proposed the incorporation of nervonic acid into functional foods to prevent inflammatory bowel conditions [27].

The examples above illustrate the significance of searching for phytonutrients rich in the aforementioned acids. However, the composition and structure of the storage triacylglycerols in the seeds of *Lunaria rediviva* have never been the subject of study. Still, it possesses a distinct advantage over *Lunaria annua* in that it is perennial, enabling it to produce a consistent annual harvest without the need for annual cultivation [28]. The present study focuses on the analysis of the fatty acid composition, the distribution of fatty acids between the *sn* positions of the carbon atom of the glycerol residue of storage TAGs, and the molecular species composition of triacylglycerols in *Lunaria rediviva*.

## 2. Materials and Methods

### 2.1. Reagents

Lipase from porcine pancreas (Sigma-Aldrich, Saint Louis, MO, USA, Cat.No L3126); *Tris*-base (Sigma-Aldrich, Saint Louis, MO, USA, Cat.No 252859); Trimethylsulfonium Iodide, 98% (Acros Organics, Geel, Belgium, Cat.No 140250250); Silver(I) oxide, 99+% (Acros Organics, Geel, Belgium, Cat.No 205800100); 2′,7′-Dichlorofluorescein, pure (Acros Organics, Geel, Belgium, Cat.No 191530050); 2,6-di-*tert*-Butyl-4-methylphenol, 99.8% (Acros Organics, Geel, Belgium, Cat.No 219830500); Trifluoroacetic anhydride, 98% (Macklin, Shanghai, China, Cat.No T818925); 2-Amino-2-methyl-1-propanol (Macklin, China, Shanghai, Cat.No A800065); Glacial acetic acid pure, (Panreac, Germany, Darmstadt, Cat.No 141008); Desoxycholic acid sodium salt (Sigma-Aldrich, Steinheim, Germany, Cat.No D6750); Acetone, 99.8% for HPLC and UV Spectroscopy (Loba Chemie, Tarapur, Maharashtra India, Cat.No 00013); Diethyl ether (Honeywell, Seelze, Germany, Cat.No 32203); 2-Propanol (Sigma-Aldrich, Steinheim, Germany, Cat.No 34863); Acetonitrile HPLC Gradient grade (Carlo Erba, Val de Reuil, France, Cat.No 412392000); *n*-Hexane, 99+%, (Acros Organics, Geel, Belgium, Cat.No AC160780025); Methanol, 99.8+% (Fisher Chemical, Trinidad and Tobago, Cat.No 11318157); Silica gel on TLC Al foils (Fluka Analytical, Steinheim, Germany, Cat.No 02599); TLC aluminum sheets, Silica gel 60 (Supelco, Darmstadt, Germany, Cat.No 1055530001); and TLC aluminum sheets, Silica gel 60 (Merck, Darmstadt, Germany, Cat.No 1168350001) were all purchased from local suppliers.

### 2.2. Plant Material

Mature silicules of *Lunaria rediviva* L. were collected at the arboretum of N. V. Tsitsin Main Botanical Garden of the Russian Academy of Sciences (location of collection: 55°50′07.6′ N, 37°36′17.4′ E) in the first decade of October 2023; the seeds (Figure S2) were subsequently isolated from the fruit in the laboratory and stored in paper bags at room temperature until the following analyses.

### 2.3. Extraction of Fatty Oil from Mature Seeds

A sample of *circa* 2 g of air-dried seeds was ground with a pestle in a porcelain mortar (*n* = 3) and then transferred into 50 mL Eppendorf-type tubes, and the fatty oil was extracted using the two-phase chloroform–methanol method [29] with the addition of 0.01% BHT. The procedure was repeated three times, and the resulting extracts were combined. Extracts were subsequently evaporated to dryness in a shaped 50 mL flask using an IKA RV-10 rotary vacuum evaporator. The residual material (a light yellow oily liquid) was weighed and redissolved in chloroform to yield a solution with a concentration of 100 mg/mL, which was stored at −20 °C until further analysis.

### 2.4. Separation of Triacylglycerols from Fatty Oil Extracts

Chromatographically pure preparations of *sn*-1,2,3-triacylglycerols were made using a two-step semi-preparative thin layer chromatography technique on 20 × 20 cm, 0.25 mm thickness Merck Silica Gel 60 plates. To this end, 1 mL of a fatty oil solution (*circa* 100 mg of oil) in chloroform was applied to the starting line using a 100 μL microsyringe, after which the plate was placed in a chamber with a mixture of *n*-hexane and diethyl ether (95:5 v/v) and left to stand for the time required for the solvent front to rise to the top edge of the plate. After this, the plate was left to dry at room temperature for 15 min and then placed in a chamber containing a second mobile phase consisting of *n*-hexane, diethyl ether, and glacial acetic acid at a ratio of 70:30:0.1 v/v/v. Once the solvent front had reached a point 15 cm from the bottom edge of the plate, the plate was left to dry at room temperature for a period of 15 min. The presence of triacylglycerols and native *sn*-1,2-diacylglycerols was indicated by UV absorption at λ = 365 nm following the spraying of the plate with a solution of 2′7′-dichlorofluorescein in methanol (80 mg/L). Silica gel get holding each lipid fraction was transferred to a glass separate glass tube using a spatula, and neutral lipids were extracted twice with 5 mL portions of chloroform. The extracts were combined and subjected to evaporation in an argon flow until dryness. The resulting preparations of TAGs and native *sn*-1,2-DAGs were dissolved in benzene to give a solution with a concentration of 10 mg/mL and stored at −20 °C until further analyses.

### 2.5. Partial Enzymatic Deacylation of Triacylglycerols

In order to obtain *rac*-1,2-DAGs and *sn*-2-MAGs from TAGs, 200 μL of a solution of TAGs in benzene (see Section 2.4) (2 mg of pure TAGs) was transferred into a 2 mL glass vial (*n* = 9). The solvent was then evaporated to dryness in an argon flow. To the TAGs in the vial, 1000 μL of a lipase suspension derived from porcine pancreas was added, which was prepared as follows. A 15 mg of dry lipase was resuspended in 5 mL of 0.25 M Tris buffer (pH 7.5). This solution was then mixed with 100 μL of 0.01 M calcium chloride solution and 250 μL of 0.1 wt% sodium deoxycholate solution (both dissolved in working Tris buffer, as described by [30]). The suspension was then shaken vigorously for one minute and subsequently added to the TAG preparations. The vials containing the lipase and TAG suspension were subjected to intense shaking for 30 s using a BioSan Microspin-2400 vortex, after which they were incubated in a solid-state thermostat at 37 °C for 35 min. Subsequently, to terminate the enzymatic reaction, 100 μL of 6M hydrochloric acid and 200 μL of ethanol were added successively to each vial. The hydrolysis products of TAGs were then extracted using two 500 μL portions of a 1:1 v/v mixture of *n*-hexane and diethyl ether. The phases were separated by centrifugation for two minutes at 2000 × G. The upper phase, containing the dissolved hydrolysis products, was collected in clean vials. The solvents were evaporated to dryness in an argon flow, and the resulting dry residue was redissolved in a 200 μL solution of *n*-hexane and diethyl ether in a 1:1 v/v for thin layer chromatography.

### 2.6. The Isolation of Pure sn-2-MAG and rac-1,2-DAG from Products of Partial Enzymatic Hydrolysis of TAGs

The hydrolysis products of triacylglycerols (*ca.* 2 mg) were applied to a 2 cm long start line on a Fluka Silica Gel 10 × 10 cm plate with three samples per plate. Once the solvent had evaporated from the plate, it was placed in a chromatographic chamber filled with a 90:10 v/v chloroform:acetone mixture as the mobile phase. The zones containing TAGs (*Rf* = 0.98), *rac*-1,2-DAGs (*Rf* = 0.74), free fatty acids (*Rf* = 0.47), and *sn*-2-MAGs (*Rf* = 0.15) were identified using dichlorofluorescein as previously described in Section 2.4. Thereafter, silica gel with *rac*-1,2-DAGs and *sn*-2-MAGs were transferred to glass vials using a spatula and lipids were extracted as previously described in Section 2.4.

### 2.7. Synthesis of Fatty Acid Methyl Esters (FAMEs) from Neutral Lipid Preparations

#### 2.7.1. Preparation of Free Fatty Acids from Neutral Lipids via Alkali Hydrolysis

A 3 mg sample of TAG was hydrolysed in 2 mL glass vials in 500 μL of 1 M potassium hydroxide solution in 80% aqueous ethanol at 70 °C for one hour. Thereafter, the alkali was neutralised with 200 μL of 20% aqueous sulphuric acid (monitored using indicator paper to ensure a slightly acidic reaction), and free fatty acids were extracted with two 500 μL portions of a 1:1 v/v mixture of *n*-hexane:diethyl ether. Subsequently, the solvent was evaporated in an argon stream, and 300 μL of a 1% solution of sulphuric acid in methanol was added to the dry residue in a vial. The vial was then maintained at a temperature of 55 °C for 30 min. After this period, 150 μL of water was added to the solution, and the fatty acid methyl esters were extracted in two 150 μL portions of *n*-hexane. The same esterification method was utilised for TAG preparations that had not undergone preliminary hydrolysis with alkali.

#### 2.7.2. Preparation of 0.2 M Trimethylsulfonium Hydroxide Solution

Trimethylsulfonium iodide (4.4 g) was dissolved by heating in 100 mL of methanol. Subsequently, 5.0 g of silver(I) oxide was added to the solution and left on a magnetic stirrer heated to 40 °C for 4 h. The methanol with dissolved trimethylsulfonium hydroxide was then filtered through a 0.45 μm syringe filter. The filtered solution was stored in a refrigerator for a maximum of six months [31].

#### 2.7.3. Preparation of FAMEs by Using 0.2 M TMSH Solution

In order to re-esterify fatty acids derived from TAGs, *rac*-1,2-DAGs, and *sn*-2-MAGs with methanol, approximately 1 mg of lipid was transferred into a 2 mL glass vial. The solvent was then evaporated to dryness in an argon stream, and 250 μL of 0.2 M trimethylsulfonium hydroxide solution in methanol was then added to the dry residue, after which the vials were capped and incubated for 30 min at 60 °C in a solid-state thermostat. Subsequently, 125 μL of a 5% aqueous acetic acid solution was added to the solution in the vials, and the fatty acid methyl esters were extracted using 250 μL of *n*-hexane. The hexane solution containing FAMEs was transferred into 300 μL of microtiter vials for GC-MS analysis [32].

### 2.8. Preparation of 4,4-Dimethyloxazoline Derivatives (DMOX) of Fatty Acids for GC-MS

In order to precisely determine the positions of the -C = C- bonds in the acyls of fatty acids, we used mass spectrometry of their DMOX derivatives, which were prepared by the following method: 3 mg of triacylglycerols, 200 μL of a 50% solution of 2-aminomethylpropanol in benzene (w/v), and 50 μL of sodium solution in methanol (320 mg of metallic sodium were dissolved in 40 mL of cold methanol, and the resulting solution was stored in the refrigerator for not more than 2 months) were added to a 2 mL glass vial. The contents of the vial were shaken vigorously, and the vial was left overnight in a dark place at room temperature, after which 500 μL of water was added to the vial, and acyl-2-methylpropanol amides were extracted with 500 μL of a 9:1 (v/v) mixture of *n*-hexane:diethyl ether. The solution was transferred to a clean dry vial, the solvent was evaporated in an argon stream, and 200 μL of trifluoroacetic anhydride was added to the acyl-2-methylpropanol amides; then, the vial was incubated for 1 h at 50 °C in a solid state thermostat. After this time, the trifluoroacetic anhydride was evaporated in an argon stream, 300 μL of water was added to the vial, and the DMOX was extracted with 500 μL of *n*-hexane. Purification of DMOX from reaction by-products was performed by thin layer chromatography on a 5 × 10 cm Merck SilicaGel 60 plate using an 80:20 v/v chloroform:acetone mixture as mobile phase. DMOX was extracted from the silica gel as previously described in Section 2.4 [33].

### 2.9. GC-MS Analysis of FAMEs and DMOXes

The capillary GC method was applied for the separation of fatty acid methyl esters. The separation was performed on an Agilent 7890A chromatograph and equipped with an Agilent 5975C quadrupole mass spectrometric detector (Agilent Technology Inc., Santa Clara, CA, USA). The chromatograph was equipped with a 60 m HP-88 capillary column with an internal diameter of 0.25 mm and a stationary phase thickness of 0.2 µm, comprising 88% cyanopropyl-12%-aryl polysiloxane. The following conditions were employed: the temperature programme was initiated at 60 °C for a period of eight minutes, followed by a gradual increase of 7 °C per minute until reaching 175 °C for a further five minutes. Thereafter, the temperature was increased at a rate of 9 °C per minute until it reached 245 °C, which was maintained for a period of 25 min. The injector temperature was set to 260 °C, and the sample was introduced with a split ratio of 20:1. Sample volume was 1 μL. The ion source temperature was set to 230 °C, the quadrupole was set to 150 °C, and the ionisation energy was set to 70 eV. Helium was employed as the carrier gas at a flow rate of 1 mL/min. The mass range scanned was 50–550 m/z.

Chromatograms were recorded using the total ion current as the measurement parameter. The peaks of methyl esters were identified by matching their chromatographic parameters (retention time, relative retention time) and mass spectra with the NIST database using Agilent MSD ChemStation v. 4.0.3 software (Agilent Technology Systems, Santa Clara, CA, USA).

### 2.10. HPLC-PDA and HPLC-MS Separations of TAGs

The composition of positional TAG species of *Lunaria rediviva* L. was analysed by non-aqueous reversed-phase high-performance liquid chromatography (HPLC) in binary gradient mode using a Shimadzu LC20-AP Prominence system, comprising two LC20-AP pumps, an SPD-M20A photodiode array matrix detector, and a MN Nucleodur C18 HTec column (250 × 4.6 mm, 5 μm). The mobile phase A was composed of 100% acetonitrile, while the eluent B was 2-propyl alcohol. The gradient programme was as follows: at the outset of the experiment, the mobile phase was composed of 100% A. Thereafter, the proportion of B was increased at a rate of 0.65% per minute until it reached 100% at the 150-min point. Between 150 and 155 min, the proportion of A was increased again until it reached 100% and kept at 100% for another five minutes until the 160-minute point. The mobile phase flow rate was 1 mL per minute, the chromatogram was recorded at a wavelength of 205 nm, and the data acquisition frequency was 0.5 Hz [34]. The triacylglycerols were dissolved in a mixture of acetonitrile, 2-propanol, and *n*-hexane with a 2:1:1 volume ratio (v/v/v) to a concentration of 10 mg/mL. The volume of sample injected was 20 μL. The resulting chromatograms were integrated and subjected to analysis in LabSolutions. The software version was 5.73 (Shimadzu Co., Tokyo, Japan). The mass fraction of each peak was determined by normalisation to the sum of the peak areas.

The same gradient programme and column were used for the HPLC-MS analysis of TAG as had been used for the HPLC-UV analysis. In order to obtain the ammonium adducts of TAGs, 1 mL of an aqueous ammonium acetate solution was added to 1 L of each mobile phase so that the final concentration was 25 mm [35]. The HPLC-MS system was an Agilent 1290 Infinity liquid chromatograph (a G4220A binary pump, a G4226A autosampler, and a G1316C thermostatted column compartment) coupled to an Agilent 6460C triple quadrupole MS detector. The electrospray ion source of the MSD operated at nebulizing gas pressure of 30 psi, while the drying gas flow and temperature were 8 L/min and 350 °C, respectively. Only positive ions were being detected. The capillary voltage was set to 3500 V, the fragmentor voltage was 100 V, and the cell accelerator voltage was 5 V. The analysis was performed both in scan mode (*m/z* 600–1200) and SIM mode (dwell time of 75 ms for each monitored ion). The acquired data were processed using Agilent MassHunter Qualitative analysis software version B.06.00 Service pack 1.

### 2.11. Calculation of Position-Type and Position-Species Compositions of Triacylglycerols

#### 2.11.1. Calculation of Fatty Acid Composition of *sn*-2, *sn*-1,3 Positions of TAG, Enrichment and Selectivity Factors

In addition to the experimental determination of the fatty acid composition in the *sn*-2 position of TAG ${\left[A\right]}_{2}$ by analysing the FA composition of *sn*-2-monoacylglycerols, which were obtained as a result of enzymatic hydrolysis, the fatty acid composition in the *sn*-2 position of triacylglycerols was calculated using the following formula [36]:

(1) $${\left[A\right]}_{2}=4\times {\left[A\right]}_{1,2(2,3)}-3\times {\left[A\right]}_{1,2,3}$$

${\left[A\right]}_{1,2(2,3)}$ represents the mass percentage of the i-th fatty acid in *rac*-1,2-diacylglycerols obtained through enzymatic hydrolysis of TAG. Similarly, ${\left[A\right]}_{1,2,3}$ denotes the mass percentage of the i-th fatty acid in triacylglycerols. Furthermore, the *sn*-1,3 position of TAGs, ${\left[A\right]}_{1,3}$ can be calculated from the fatty acid composition of *sn*-1,2,3-TAGs, ${\left[A\right]}_{1,2,3}$, and their *sn*-2 position ${\left[A\right]}_{2}$, which in turn can be calculated using Formula (1) or found experimentally (as described in Section 2.6). For the calculation, we used the following formulas [36]:

(2) $${\left[A\right]}_{1,3}=\frac{3\times {\left[A\right]}_{1,2,3}-{\left[A\right]}_{2}}{2}$$

(3) $${\left[A\right]}_{1,3}=3\times {\left[A\right]}_{1,2,3}-2\times {\left[A\right]}_{1,2(2,3)}$$

In order to characterise incorporation of major unsaturated FAs into the *sn*-2 position of TAGs, enrichment factors (EFs) and selectivity factors (SFs) were calculated as follows [36]:

(4) $$EF=\frac{{\left[A\right]}_{2}}{{\left[A\right]}_{1,2,3}}$$

(5) $$SF=EF\times \frac{{\left[U\right]}_{1,2,3}}{{\left[U\right]}_{2}}$$

#### 2.11.2. Calculation the Position-Species Composition of TAGs Using the Vander Wal Model

In order to calculate the positional-species composition of TAGs, which encompasses a range of individual FA residues (*a*, *b*, *c*, and so forth), the following equations were employed [36]:

(6) $$\begin{array}{cc} \left[aaa\right] & ={\left[a\right]}_{1,3}^{2}\times {\left[a\right]}_{2}\\ \left[aba\right] & ={\left[a\right]}_{1,3}^{2}\times {\left[b\right]}_{2}\\ \left[aab\right] & =2{\left[a\right]}_{1,3}\times {\left[a\right]}_{2}\times {\left[b\right]}_{1,3}\\ \left[abc\right] & =2{\left[a\right]}_{1,3}\times {\left[b\right]}_{2}\times {\left[c\right]}_{1,3} \end{array}$$

The calculations were performed in accordance with the Vander Wal model utilising the UTCA programme, which was developed for this purpose [37].

### 2.12. Statistical Analysis

Unless otherwise stated, all experimental data are presented as arithmetic mean values (±SD) obtained from three biological replicates (each with three analytical replicates) for one lipid fraction. The statistical analysis was conducted using the SigmaPlot 14.0 software package (Systat Software Inc., San Jose, CA, USA). The significance of the differences between the datasets was analysed using a one-way analysis of variance with the Holm–Sidak test for all pairwise multiple comparisons post hoc.

## 3. Results

### 3.1. Optimisation of a Lipid Derivatisation Method for Fatty Acid Analysis

During the preparation of FAMEs from TAGs by their direct transesterification in 1% methanolic $H_{2}SO_{4}$ solution at 55 °C (see Section 2.7.1), we noticed that the reaction did not proceed quantitatively. A drop of unreacted TAGs remained at the bottom of the reaction vials. Obviously this was due to the presence of a large number of molecular species of TAGs with very long-chain unsaturated FAs in their composition, which have a higher melting point compared to TAGs of the typical composition. Therefore, we also applied other common sample preparation methods to the TAGs from the seeds of *Lunaria rediviva* and matched influence of sample preparation on the observed FA composition. The results are shown in Table 1.

As can be seen from the data in Table 1, the FA compositions of the FAMEs obtained by the classical method via saponification to free fatty acids with subsequent re-esterification were completely identical to those of FAMEs obtained by the one-step method with trimethylsulfonium hydroxide (TMSH). Both methods of preparing FAMEs gave significantly higher yields of oleic and nervonic acids than the protocol of direct re-esterification of TAGs with 1% sulfuric acid in methanol. In the light of these data, we decided to abandon direct re-esterification methods (due to the non-quantitative process) and saponification, which is a time-consuming method, in favour of esterification with trimethylsulphonium hydroxide. This method allowed quantitative and adequate data to be obtained, with the derivatisation of TAGs to FAMEs being carried out quantitatively in only 30 min in a single-step reaction.

### 3.2. Fatty Acid Composition of Triacylglycerols from Mature Seeds of Lunaria rediviva L.

Using GC-MS with a 60 m HP-88 capillary column with a highly polar stationary phase, we detected 20 individual C16–C24 fatty acids, both saturated, mono-, and polyunsaturated, in the TAG composition of the seeds.

**Figure 1:**
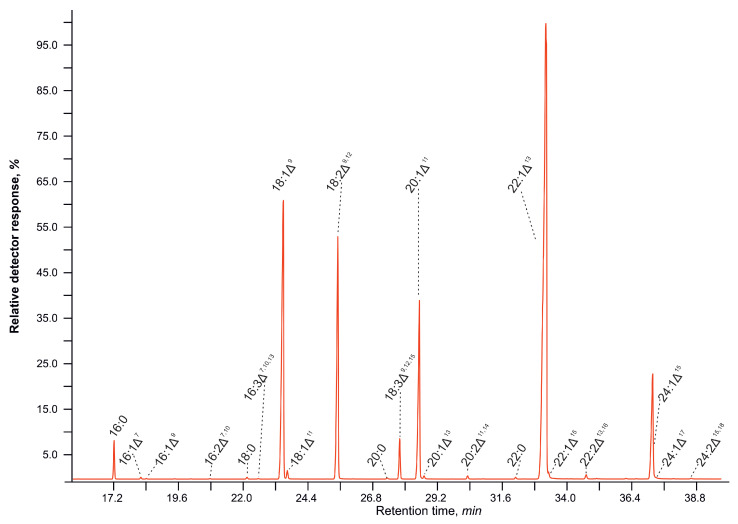
A gas chromatography–mass spectrometry (GC-MS) chromatogram of fatty acid methyl esters derived from triacylglycerols of mature seeds of *Lunaria rediviva* L. (total ion current).

Figure 1 shows the result of the chromatographic separation of fatty acid methyl esters from triacylglycerols of Lunaria ridiviva. It can be seen that the major fatty acids detected were oleic, linoleic, gondoic, erucic, and nervonic acids, representing a proportion of over 93% of the total fatty acids. The remaining minor acids present in the TAG were palmitic, hexadecenoic, hexadecatrienoic, *cis*-vaccenic, arachidic, eicosadienoic, behenic, docosenoic, docosadienoic, tetracosenoic, and tetracosadienoic acids.

Mass spectrometry of their DMOX derivatives was employed to determine the precise positions and quantities of ethylene bonds in fatty acid acyls derived from the seed’s triacylglycerols.

**Figure 2:**
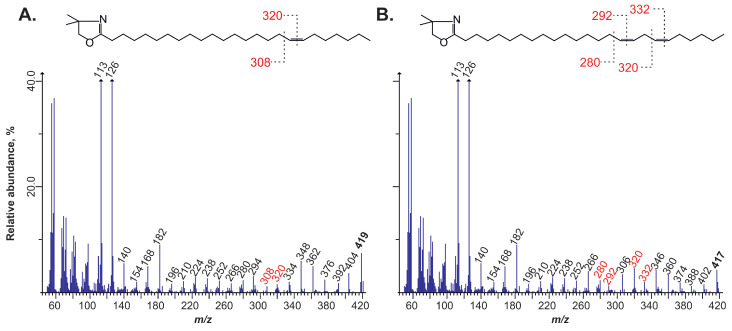
Mass spectra of DMOX derivatives of unsaturated C24 fatty acids. (Panel **A**) depicts the spectrum of *cis*-17-tetracosenoic acid, while (panel **B**) depicts the spectrum of *cis*,*cis*,-15,18-tetracosadienoic acid.

Figure 2 depicts the DMOX mass spectra of the DMOX derivatives of unusual isomers of unsaturated C24 acids.

The mass spectrum in Figure 2A is characterised by the prevalence of fragment masses of m/z = 113 (McLafferty ion) and m/z = 126. These ions are formed by the fragmentation of the heterocycle at the carbon atom with the carboxyl group of the DMOX derivative. Subsequently, a series of fragment masses differing by 14 amu (m/z = 140, 154, and so on) was observed, indicating the “detachment” of the methylene (-CH_2_-) groups of the aliphatic chain. This sequence continued until a pair of fragment masses, m/z = 308 and 320, differing by 12 amu, was reached. This indicates the loss of a hydrogen atom at two carbon atoms, which is indicative of the presence of an ethylene bond. From the fragment mass m/z = 320, a series of ions differing by 14 amu resumes, a molecular ion with m/z = 419, was preceded by the fragment mass m/z = 404. The 15 amu difference between these ions indicates the presence of a terminal -CH_3_ fragment, which corresponds to a DMOX derivative of a fatty acid with one ethylene bond and 24 atoms in the aliphatic chain. Therefore, based on the aforementioned evidence, it can be concluded that the ethylene bond is situated between the C17 and C18 atoms. Consequently, the identified fatty acid can be determined to be 24:1Δ17.

Figure 2B illustrates that the molecular ion has a mass of m/z = 417, which is two amu units less than that of DMOX 24:1 fatty acid. This indicates the presence of two ethylene bonds in its structure. Ions with a mass difference of 12 amu are m/z = 280–292 and m/z = 320–332, indicating the position of double bonds between 15 and 16 and 18 and 19 carbon atoms in the aliphatic chain. Therefore, the spectrum belongs to the DMOX derivative of methylene-interrupted dienoic 24:2Δ15,18 fatty acid.

This approach allowed us to identify all the unsaturated fatty acids present in the triacylglycerols of the mature seeds of *Lunaria rediviva*. The structure, bond positions, and diagnostic ions of their DMOX derivatives are summarised in Table 2.

Based on the established positions of ethylene bonds in a series of isomers of mono- and polyunsaturated fatty acids, it can be concluded that during their biosynthesis, there are processes of Δ9-desaturation of 16:0 and 18:0 acyls to hexadecenoic and octadecenoic acids and a series of successive C2-elongations to form monounsaturated fatty acids with very long chains. Thus, gondoic acid is formed from oleic acid by C2-elongation, and from it, in turn, erucic acid and then nervonic acid are formed. The same is true for elongation of di- and polyunsaturated fatty acids.

### 3.3. Quantitative Composition of Fatty Acids of sn-1,2,3-, sn-2-, and *sn*-1,3-Positions of Triacylglycerols of Mature Lunaria rediviva Seeds

Table 3 demonstrates the fatty acid compositions of various *sn* positions of storage triacylglycerols derived from the seeds of mature fruits of *Lunaria rediviva* L. It can be seen that the triacylglycerol composition (*sn*-1,2,3 positions) is characterised by the presence of five major fatty acids, namely, oleic, linoleic, gondoic, erucic, and nervonic, as well as a smaller proportion of palmitic, stearic, and linolenic acids. The proportion of unsaturated FA exceeded 96% of the total fatty acids, indicating that the triacylglycerols in question belong to the UUU type. This implies that they contain unsaturated FA in all three *sn* positions. It is also noteworthy that the ratio of the sum of 18:1–18:2–18:3 fatty acids to the sum of 20:1–24:1 is almost equal to 1:2, at 36.1:60.6 mass.-%. This allows us to hypothesise that a significant number of symmetrical ABA species can be expected in the composition of the molecular types of TAGs, where A and B are acyls of very long-chain fatty acids (VLCFAs) and unsaturated C18 acids, respectively.

The *sn*-2 position compositions of TAGs, as calculated using the Vander Wal model, exhibited identical sets of fatty acids in very close mass ratios, a finding that was consistent with those observed in the experimental data. Therefore, the results of the ANOVA tests indicated that there were no statistically significant differences in the mass fractions of oleic, linoleic, or linolenic acids in the *sn*-2 positions between the experimental and calculated data. In both instances, the total amount of unsaturated C18 acids constituted 99.4% (in *sn*-2-monoacylglycerols obtained through enzymatic hydrolysis) and 94.3% in the calculated *sn*-2 position.

A comparable phenomenon was observed with regard to the structure of the *sn*-1,3 positions. The calculations performed using *rac*-1,2-DAGs and *sn*-2-MAGs obtained through the enzymatic hydrolysis of TAGs yielded values for the mass fractions of fatty acids that were found to be in close consistency. It can be observed that unsaturated C18 acids were almost entirely absent from the *sn*-1,3 positions, with these positions being acylated by 96.4% monounsaturated C20–C24 acids. The remaining 0.6% were palmitic and stearic acids. It can thus be seen that the TAGs of *Lunaria rediviva* seeds displayed a markedly positional specificity of structure, which was characterised by a distinct preference for the inclusion of 18:1–18:3 acids in the *sn*-2 position and an exclusive acylation of the *sn*-1,3 positions by saturated acyls and monounsaturated C20:1–C24:1 FAs.

Table 4 illustrates the enrichment factors for the major fatty acids present in the triacylglycerol composition of *Lunaria rediviva* seeds. It can be noticed that the EF values for 18:1–18:3 were within the range of 2.5–3.72, which serves to reinforce the hypothesis that these acids exhibit a preference for the *sn*-2 position of TAG during the biosynthesis process. The indicator for 20:1–24:1 is zero. A comparable pattern is evident in the selectivity factor values.

Therefore, it can be concluded that the structure of storage triacylglycerols (TAGs) in the oil of mature *Lunaria rediviva* seeds is characterised by a strict positional specificity. In the light of this observation, it was of great interest to characterise the composition of the molecular species of TAGs. For this purpose, the Vander Wal model and non-aqueous reversed-phase HPLC with UV detection at 205 nm were employed for the calculation method; the same HPLC mode was subsequently used for TAG identification with an MS detector (electrospray ionisation).

### 3.4. Positional-Species Composition of Storage Triacylglycerols from Mature Seeds of Lunaria rediviva

#### 3.4.1. Qualitative Composition of TAG Molecular Species Determined by Non-Aqueous Reversed-Phase HPLC

**Figure 3:**
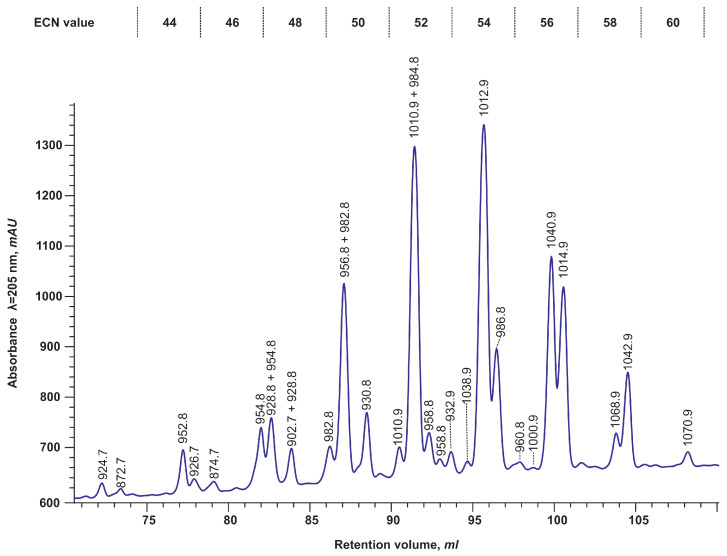
HPLC-UV λ = 205 nm chromatogram of separation of a mixture of triacylglycerol molecular species from mature seeds of *Lunaria rediviva* and the corresponding predominant compound masses in each peak (by ESI-MS analysis).

One of the most frequently employed and established technique for the analysis of triacylglycerol mixtures is non-aqueous HPLC on a reversed C18 phase, utilising a range of detectors, including PDA, ELSD, CAD, and MS. The analysis of *Lunaria rediviva* TAGs was conducted using the method proposed by [34] with minor modifications. To achieve elution of all molecular species containing C20:1–C24:4 fatty acids, the gradient had to be increased to 150 min, during which time the proportion of 2-propanol in the mobile phase reached 100%. The results of the separation with UV detection at λ = 205 nm are presented in Figure 3.

The chromatogram displays the peaks comprising different TAG molecular species eluted in a series of peaks in accordance with their equivalent carbon number (ECN) defined as CN (number of carbon atoms in a TAG molecule) minus ND (number of ethylene bonds in the structure of fatty acid acyl groups). The mean time required for a 2 ECN unit step was 6 min.

**Figure 4:**
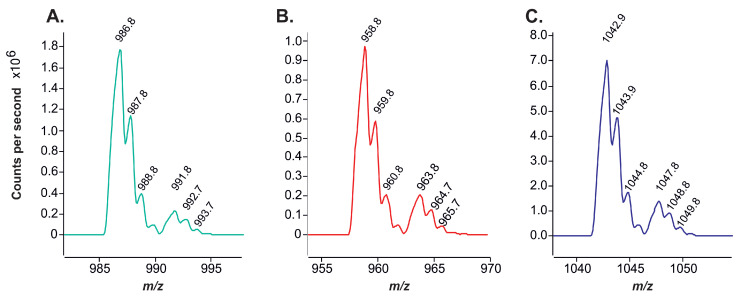
Mass profile of individual peaks of different molecular species of TAGs belonging to ABC and ABA types (**A**)—*rac*-20:1–18:1–22:1, ECN = 54; (**B**)—20:1–18:1–20:1, ECN = 52; (**C**)—*rac*-22:1–18:1–24:1, ECN = 58.

To identify the TAG molecular species in each peak, the analysis was repeated in the same mode but on an instrument with an ESI-MS detector. This enabled the acquisition of a mass profile for each peak. Figure 4 exhibits representative mass profiles of individual peaks, including various triacylglycerol molecular species. It can be seen that the most prevalent ions formed by the studied TAGs were ${\left[M+NH_{4}\right]}^{+}$, while sodium adducts were also identified with the corresponding signal intensities approximately five-fold lower.

The strict positional specificity of the storage triacylglycerols of *Lunaria rediviva* enabled the identification of the main molecular types of TAGs based solely on the mass of their adducts with ammonium, ${\left[M+NH_{4}\right]}^{+}$. To illustrate, the mass of 1042.9 m/z can be exclusively ascribed to the TAG of the ABC type, which should contain (in accordance with the previously identified fatty acids) acyls of 22:1, 18:1, and 24:1 FAs. The results of the enzymatic deacylation indicated that the *sn*-2 position is exclusively acylated by unsaturated C18 fatty acids. Consequently, in TAGs with a molecular weight of 1042.9 m/z, the *sn*-1,3 positions were acylated by 22:1 and 24:1 fatty acids, respectively. Given these observations, the peak in question is presumed to contain a racemic mixture of *sn*-1-erucoyl-2-oleyl-3-nervonoyl-glycerol with *sn*-1-nervonoyl-2-oleyl-3-erucoyl-glycerol or *rac*-22:1-18:1-24:1.

Thus, we were able to identify all the main peaks of the molecular species of TAGs through a comparison of the fatty acid composition analysed at different *sn* positions of TAGs with the elution order of TAGs and with the mass of their ammonium adducts under reserved-phase non-aqueous HPLC conditions.

#### 3.4.2. Quantitative Composition of Molecular Species of TAG, Experimental Data Compared with the Calculation Method

One of the convenient method for describing the composition of triacylglycerols without strict identification of their molecular species is the grouping of peaks according to their equivalent carbon numbers (ECNs) [38].

**Figure 5:**
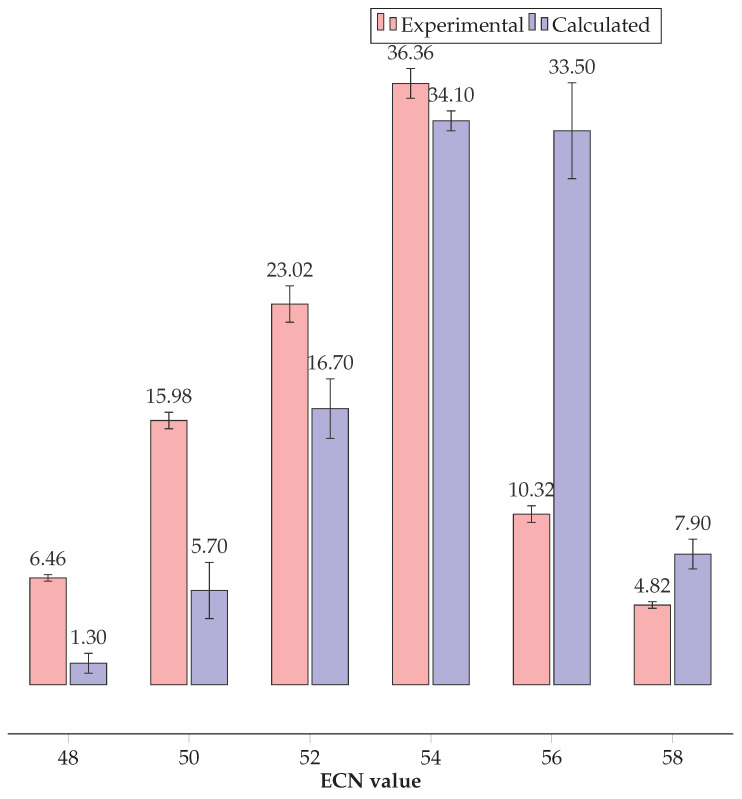
Fractional composition of triacylglycerols grouped by ECN value found experimentally and calculated using the Vander Wal model, where mol.% (*n* = 9).

As previously stated, under reversed-phase non-aqueous HPLC conditions, a series of peak groups were observed in 6-minute intervals (Figure 3) corresponding to an increase in the ECN by two units. Despite the differences in the composition of fatty acids of the molecular species of TAGs, each peak group had its own ECN value. Figure 5 illustrates the composition of TAGs, as observed experimentally and calculated in accordance with the Vander Wal model.

The Vander Wal model of limited statistical distribution of fatty acid acyl groups in the case of TAGs from *Lunaria rediviva* provides a satisfactory prediction of the composition of the ECN groups of triacylglycerol molecular species.

The model thus exhibited a high degree of accuracy in predicting the molar fractions of TAGs with ECN = 54, as well as in calculating the fraction of TAGs with ECN = 52 and ECN = 58, which were found to be in close agreement with the experimental results. The greatest discrepancies between the model and the experimental results were observed for TAGs with ECN = 48–50 and 56, with a difference of 2.8–5.0 times the experimental value between the calculated and observed quantities. Therefore, in the case of TAGs with ECN = 48–50, the model exhibited an underestimation of the expected amounts of TAGs by approximately 3–5 times, whereas for TAGs with ECN = 56, it demonstrated an overestimation of the amount by nearly 3 times in comparison to the experimental data.

One method for illustrating the extent of acid affinity for the *sn*-2 position of triacylglycerols is to calculate and compare the enrichment factors (EFs). The selectivity factor SF enables a comparative evaluation of such affinity in TAGs with varying degrees of unsaturation [39]. The enrichment factor (EF) is defined as the ratio of the molar concentration of the acyl group at the *sn*-2 position to its concentration in the total amount of triacylglycerol (TAG). The selectivity factor (SF) is defined as the enrichment factor (EF) of a specific fatty acid (FA) divided by the EFs of all FAs that are preferentially esterified at the *sn*-2 position [40]. The value of the enrichment factor (EF) can range from 0 to 3, with values less than 1.0 indicating a preference for the *sn*-1 and *sn*-3 positions and values greater than 1.0 indicating a preference for the *sn*-2 position [40]. The EF concept is also useful when comparing the values of FAs competing for the *sn*-2 position in the same oil. However, it is less convenient for discussing the behaviour of FAs in several different oils. For this reason, Gunstone et al. proposed the SF, which compensates for some of the existing difficulties and makes comparisons between oils easier and more meaningful [40].

#### 3.4.3. Peculiarities of the Positional-Species Composition of TAGs of *Lunaria rediviva* Seeds

As previously stated, a notable divergence was observed between the theoretical calculations based on the *sn*-1,3-random-2-random acyl distribution model and the experimental findings at the group composition level of TAGs according to their ECN value. It was of significant interest to elucidate the positional-species composition of triacylglycerols at the level of the main molecular species.

**Figure 6:**
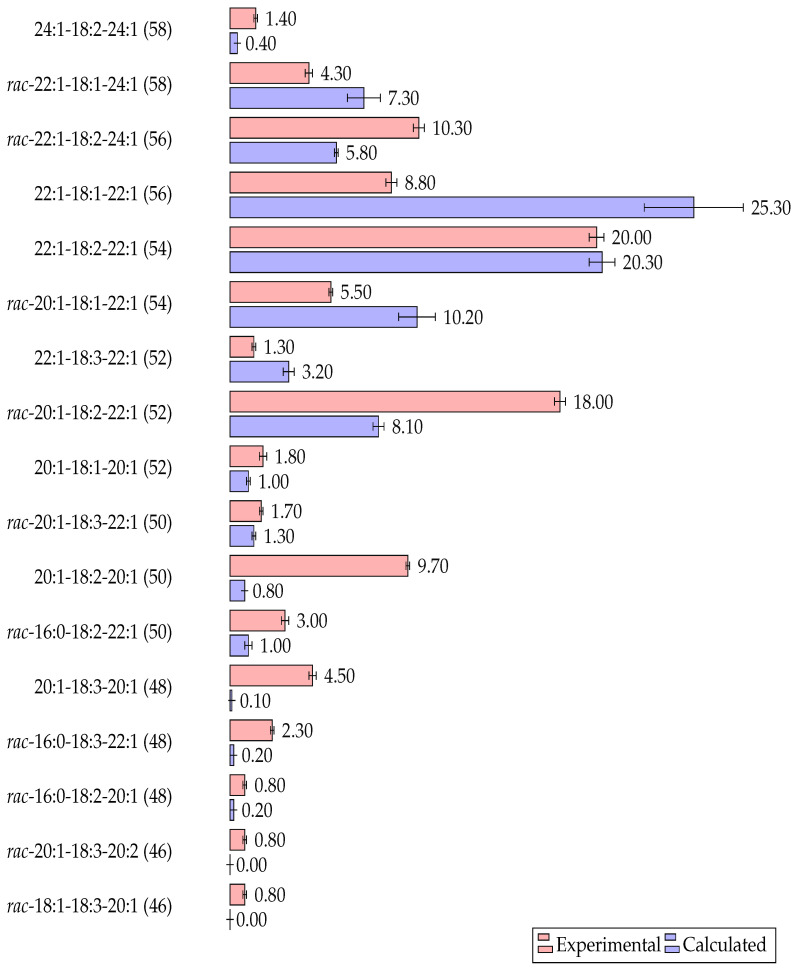
Positional-species composition of triacylglycerols of *Lunaria rediviva* determined by the NA-RP-HPLC method and calculated using the Vander Wal model.

Figure 6 illustrates the positional species composition of the TAGs, both those observed experimentally and those calculated in accordance with the Vander Wal theory. The molecular species were ordered according to their decreasing ECN value, and only those with a molar fraction exceeding 0.8% are presented.

It can be observed that the sum of 17 individual positional species of triacylglycerols in the experimental results accounted for 95% of all TAGs. The sum of the same species, for which concentrations were calculated, accounted for 85.5%. In other words, the model as a whole not only predicts the typical or group composition of TAGs but also the expected set of their positional types.

In accordance with the Vander Wal model, the proportion of TAGs of the ABC and ABA types (where A, B, and C are fatty acid acyls) was approximately 30 and 60%, respectively. This indicates that they were formed in a ratio of 1:2. Conversely, the experimentally determined ratio between triacid (ABC) and diacid (ABA) TAGs was almost equal to 1:1.

It can thus be concluded that there is not only positional discrimination with regard to the inclusion of acyls of monounsaturated C20-C24 fatty acids in the *sn*-2 position of TAG but also, apparently, competitive acylation of *sn*-1,2-diacylglycerols with disparate unsaturated C18 acids in the *sn*-2 position and monounsaturated C20:1–24:1 acids in the *sn*-1 position.

This observation is consistent with the findings made using of Vander Wal’s statistical model, which indicates that the proportion of *sn*-1,3-dierucoyl-2-oleoyl-glycerol in the oil of mature seeds of *Lunaria rediviva* should be at least 25%. However, the actual proportion is no more than 9%, indicating a discrepancy of 2.8-fold with the model. In contrast, the calculated value for the triacid *rac*-1-gondoyl-2-linoleoyl-3-erucoyl-glycerol is 2.2 times less than the experimental value, with the respective percentages being 18% and 8.1%.

It is also noteworthy that nervonic acid exhibited a similar distribution pattern, being predominantly present in triacid molecular species and associated primarily with those containing linoleic acid in the *sn*-2 position. Therefore, dinervonoyl-triacylglycerol was solely represented by the *sn*-1,3-nervonoyl-2-linoleyl glycerol species, accounting for 1.4% of the total, while the combined share of triacid TAGs containing 24:1 acyls was 14.6%, of which *rac*-1-erucoyl-2-linoleyl-3-nervonoyl accounted for 10.3%.

With regard to triacylglycerols containing α-linolenic acid in the *sn*-2 position, it is notable that molecular species with gondoic acid were particularly prevalent. Therefore, of the 9.5% of all TAGs with 18:3 acid in the *sn*-2 position, 6.2% were *sn*-1,3-digondonoyl-2-linolenoyl-glycerol and *rac*-gondonoyl-2-linolenoyl-3-erucoyl-glycerol in quantities of 4.5 and 1.7%, respectively.

## 4. Discussion

### 4.1. An Investigation into the Features of Fatty Acid Biosynthesis in Lunaria rediviva Seeds

Three enzyme systems have been identified as the catalysts for the biosynthesis of fatty acids in higher plants, operating sequentially until the target acyl is formed [41]. At the initial stage, which predominantly occurs in plastids, the participation of acetyl-CoA carboxylase (ACC) and fatty acid synthase (FAS) is essential for the biosynthesis of saturated C16 or C18 acyls. These acyls, in a complex with the acyl-carrying protein, undergo the first desaturation. The subsequent action of stearoyl-acyl-ACP desaturase results in the formation of 18:1Δ9-ACP from 18:0-ACP. This is then transported from the plastid to the endoplasmic reticulum with the assistance of integral proteins from the FAX (fatty acid export) family [42,43]. Subsequently, in the cytosol and ER, the fate of 18:1 acyl develops along the so-called eukaryotic pathway, during which it can be desaturated to 18:2Δ9,12 and 18:3Δ9,12,15 fatty acids, or 18:1 acyl can become a substrate for the elongases. Elongases are enzymes that "build up" the length of saturated and unsaturated fatty acids by adding two methylene groups to them. These enzymes have been the subject of extensive research, with their substrate specificity being a key area of study [44].

Elongase, the enzyme responsible for the formation of monounsaturated fatty acids in another species of the genus *Lunaria*—annual honesty—has been the subject of a detailed study. Eberhard Fehling and Kumar D. Mukherjee were able to isolate a membrane fraction from the cotyledons of maturing seeds of *Lunaria annua* by centrifugation at 15,000 G. They demonstrated that this fraction was capable of synthesizing thioesters of very long-chain acyl-CoA. Furthermore, the mechanism of action of the enzyme in question was found to be identical to that of analogous enzymes in mammals [45,46]. The process of elongation of unsaturated acyl groups occurs by the condensation of acyl-CoA with malonyl-CoA to form β-ketoacyl-CoA, which is then reduced to β-hydroxyacyl-CoA. This intermediate then undergoes dehydration to *trans*-2-enoyl-CoA, and this product is finally reduced to acyl-CoA, which is elongated by two methylene groups compared to the original substrate [46]. The elongation process is NADH/NADPH-dependent. The authors also observed that acyl-CoA elongase from *Lunaria annua* exhibited no significant substrate specificity for any of the saturated (14:0–20:0) or omega-9 monounsaturated (14:1–22:1) acyl-CoA substrates. However, they noted that C18–C20 substrates were elongated at a slightly faster rate compared to others [46]. Nevertheless, the authors did not consider diunsaturated acids in their research.

The data obtained from the current study of the fatty acid composition of perennial honesty lipids are, in general, in excellent agreement with the conclusions on substrate specificity made by previous researchers [45,46]. In the present study, a method was employed for highly accurate identification of the position of ethylene bonds in fatty acid acyls based on mass spectrometry of their DMOX derivatives [47]. This method enabled the study of the biosynthesis and elongation of fatty acid acyls. Indeed, in *Lunaria rediviva*, C2 elongation of monounsaturated acyl occurs, and elongase apparently does not have substrate specificity for the position of the ethylene bond within the acyl, which can be judged by the presence of minor products of very long-chain unsaturated acids formed from cis-vaccenic acid. The following series of minor fatty acids was observed: 18:1Δ11→20:1Δ13→22:1Δ15→24:1Δ17. It is noteworthy that the perennial honesty elongase exhibited a high degree of specificity for monounsaturated acyls, as evidenced by the negligible elongation of linoleic acid, with only trace amounts of its elongation products being detected: 18:2Δ9,12→20:2Δ11,14→22:2Δ13,16→24:2Δ15,18. The elongation products of 18:3Δ9,12,15 acid were completely absent. Consequently, it can be hypothesised that the active site of the enzyme is capable of binding exclusively to monounsaturated acyl groups in which the ethylene bond is located at a distance of nine (and, to a lesser extent, seven) carbon atoms from the aliphatic chain’s methyl end. It is evident that the geometry of the acyl plays a pivotal role in determining substrate specificity; however, this supposition necessitates further experimental validation.

### 4.2. On the Structure of Storage TAGs Containing a Large Number of Very Long-Chain Monounsaturated Fatty Acids

The vast majority of plant cell lipids consist of lauric (12:0), myristic (14:0), palmitic (16:0), stearic (18:0), oleic (18:1Δ9), linoleic (18:2Δ9.12), and α-linolenic (18:3Δ9.12.15) acids. These seven fatty acids, which possess an even number of carbon atoms in their aliphatic chains, are prevalent in natural biological systems and, as a consequence, have been designated as the *main* fatty acids. Concurrently, the qualitative composition of FAs in diverse lipids can exhibit substantial variability. For instance, the composition of plant triacylglycerols typically contains FAs that are absent in the membranes of their corresponding organisms. Notably, the triacylglycerols present in storage organs, such as seeds and the juicy, oil-bearing, non-seed parts of fruits, have been found to contain more than 1000 distinct types of fatty acids [41].

Fatty acids that are homologous to the major fatty acids but are present in lipids in smaller quantities (no more than 5% of the total FAs) are classified as *minor* FAs. Minor FAs also include homologs of the major FAs with a greater or lesser number of carbon atoms or differ from the major FAs in the position or configuration of double bonds. It is notable that both major and minor FAs are present in the lipids of the vast majority of plants; as such, they are termed *usual* FAs. The remaining acids, which differ from them either in the configuration or position of double bonds, or in the presence of conjugated double or triple bonds, and which are also found in lipids, are classified as unusual. For instance, more than 1000 different unusual FAs have been identified in the composition of plant seed oils [41], although it is hypothesised that several thousand more unusual FAs may be present in the plant kingdom [48]. It has been assumed that higher plants accumulate unusual acids in reserve acylglycerols with a view to preventing the acylation of membrane lipids by such acids. These acids are important for the normal vital activity of plant cells, the formation of responses to temperature, and other biotic and abiotic factors [49].

In the context of the aforementioned classification of fatty acids, eicosenoic, docosenoic, and tetracosenoic *n*-9 acids, which are components of TAGs, can be regarded as both *unusual* and *major* at the same time. Consequently, their *n*-7 isomers of the ethylene bond position can be classified as minor acids. As demonstrated above, these acids exclusively acylate the *sn*-1 and *sn*-3 positions, accounting for 60% of all fatty acids. Conversely, the predominant acids in the *sn*-2 position were constituted exclusively by the usual 18:1–18:2–18:3 acids. This finding is in full agreement with the observations of other researchers who studied highly erucic varieties of rapeseed, mustard [5,50], and other plants whose seed oils contains many monounsaturated fatty acids with a very long chain, for example, in some species of maple [51] or *Lunaria annua* [6,52]. Consequently, the hypothesis of a limited statistical distribution of fatty acid acyls during the biosynthesis of triacylglycerols can be proposed. It is noteworthy that this distribution pattern is not exclusive to plants within the Brassicaceae family but extends to those that are phylogenetically distant, such as plants belonging to the genus *Acer*.

### 4.3. Peculiarities of the Positional-Species Composition of TAGs in Perennial Honesty Oil

The molecular species of TAGs formed by a set of fatty acids acylating all three carbon atoms of the glycerol backbone can be predicted with a high degree of probability (for some objects with a high degree of accuracy [53]). This conclusion is based on statistical models that logically follow from the expansion of Newton’s binomial. Several models were created, and they can be categorised into two distinct groups: the first group encompasses models that represent the statistical distribution of acyl groups within the TAG molecule, witha notable example being the Frank Gunstone model. The second group comprises models that declare a constrained statistical distribution, accounting for the intricacies associated with the discrimination of specific fatty acids within the designated *sn* positions of the carbon atom of the glycerol residue. An example of this is the *sn*-1,3-random-2-random acylation model proposed by Vander Wal.

The model is predicated on the assumption that *sn*-1,3 positions are acylated from one pool of fatty acids, and the *sn*-2 position can be acylated from another set of fatty acids. It was derived from empirical observations of the structural features of the TAGs in plant oils. It is well established that saturated acids in higher plants invariably exclusively acylate the *sn*-1,3 positions, and the *sn*-2 position of the TAGs is invariably occupied by unsaturated and polyunsaturated C18 acids [41]. The model has been successfully applied to calculate the positional-species composition of the TAGs in a variety of plant oils, including those of classical oil crops such as olive and sunflower [53], as well as more specific cases, such as the description of sea buckthorn oils [39] and *Euonymus* species [36]. It has also been used to study the kinetics of changes in the positional-species composition of the TAGs in some oil plants [39].

Furthermore, cases of experimentally detected deviations from the Vander Wal model allow hypotheses to be formulated about the existence of alternative pathways for the formation of triacylglycerols, as was done using the example of sea buckthorn hypanthium oil [54]. Consequently, the authors drew attention to the accumulation of palmitic acid in the *sn*-2 position of the TAGs of sea buckthorn hypanthiums of the Caucasian climatype (up to 11%, which was almost three times greater than the 3% “deviation” allowed by the model for the sum of saturated acids in the *sn*-2 position). This phenomenon was exclusively observed in the composition of the TAGs of sea buckthorn hypanthiums, while in the seeds TAGs, the distribution of fatty acid acyls fully corresponded to the initial postulates of the model. Consequently, the authors observed the formation of molecular species of TAGs that are atypical for vegetable oils, primarily tripalmitin. This observation led to the assumption of the presence of various TAG biosynthesis systems in this plant [54].

It is unfortunate that such cases are so infrequent. Consequently, in order to detect deviations from the model, it is necessary to employ complex methods of instrumental analysis. For example, RP-HPLC with mass spectrometry can be utilised to identify the molecular species of TAG.

As previously mentioned, the sum of monounsaturated C20–C24 fatty acids in the composition of *Lunaria rediviva* oil accounted for almost 60% of the sum of fatty acids in the TAG, and the ratio of the sum of these FAs to the sum of unsaturated C18 acids was equal to 2:1. It can be reasonably assumed that symmetrical diacid triacylglycerols of the ABA type should prevail in the composition of *Lunaria rediviva* oil. The Vander Wal model calculates that they should account for 60% of all TAGs, while triacid TAGs account for the remaining 30 percent (i.e., the ratio should be 2:1).

However, experimental findings revealed an ABA/ABC TAG ratio of 1:1.5. This discrepancy can be attributed to an overestimation of the content of specific positional types of TAGs, such as *rac*-22:1–18:2–24:1 and *rac*-20:1–18:2–24:1, as observed in the experimental results. Conversely, the content of ABA type TAGs, exemplified by 22:1–18:1–22:1, 20:1–18:1–20:1 and 20:1–18:3–20:1 as determined by the Vander Wal model, was found to exceed the experimental values by a factor of 2–3. This discrepancy between the experimental findings and the statistical model raises a valid question regarding the underlying causes of this phenomenon. It has been observed that certain isoforms of enzymes specific to the acyl length and positional fatty acid composition of diacylglycerols are present in plants of the genus *Lunaria*. These enzymes have been shown to participate in the biosynthesis of triacylglycerols via the Kennedy pathway [41].

As is known, the biosynthesis of triacylglycerol in higher plants via the acyl-CoA-dependent pathway, also known as the Kennedy pathway, occurs in three stages, during which sequential acylation of the *sn*-1, *sn*-2, and *sn*-3 positions of the carbon atom of the glycerol residue with fatty acids occurs, and this process is strictly stereospecific [41,55]. The acylation of the *sn*-1 position of glycero-3-phosphate is catalysed by *sn*-glycerol-3-phosphate-acyltransferase (GPAT), resulting in the formation of lysophosphatidic acid. This, in turn, is a substrate for lysophosphatidic acid acyltransferase (LPAAT). This enzyme is responsible for the acylation of the *sn*-2 position of lysophosphatidic acid, resulting in the formation of phosphatidic acid, which, under the action of phosphatidic acid phosphatase, is converted into *sn*-1,2-diacylglycerol. Native diacylglycerols are substrates for many enzymes involved in the biosynthesis of both polar (membrane) and neutral lipids. The DAG acyltransferase (DGAT) family is responsible for the formation of triacylglycerol from *sn*-1,2-diacylglycerols [41].

These enzymes are characterised by the presence of two distinct binding sites for different substrates. The first site is responsible for binding to an acyl-containing lipid, and the second for binding to acyl-CoA [56]. If at least one binding site possesses a specific geometry, for example, to the length of the acyl in the lipid or acyl-CoA, then the enzyme begins to exhibit substrate specificity to the acyl, which most fully corresponds to the geometry of the active centre of the enzyme binding site. This assumption gives rise to a logical conclusion, namely, that plants capable of accumulating triacylglycerols with monounsaturated fatty acids with a very long chain in the *sn*-1 and *sn*-3 positions must have at least two special isoforms of enzymes involved in the biosynthesis of such TAGs. The first of these enzymes is *sn*-glycerol-3-phosphate-acyltransferase (GPAT), which is specific to acyl-CoA with C20:1–24:1 fatty acids. The second enzyme, DGAT, should exhibit specificity not only to acyl-CoA with C20:1–24:1 fatty acids but also to *sn*-1,2-diacylglycerols containing C20:1–24:1 acyl in the *sn*-1 position.

A recently discovered isoform of lysophosphatidic acid acyltransferase (LPAAT) specific to myristic acid, which belongs to the group of medium-chain fatty acids, was identified in the *Cyanobacterium sp*. IPPAS B-1200 strain [57]. As previously mentioned, phosphatidic acid can be utilised by the cell for the synthesis of polar lipids and for the production of substrates, *sn*-1,2-diacylglycerols, which are precursors of TAGs [2,41]. In contrast, relatively recently, a distinct case was observed in plants from the genus *Euonymus*, where a unique DGAT with an exceptionally unconventional substrate specificity was identified. The initial substrates for this enzyme were found to be *sn*-1,2-diacylglycerols, which are acylated with fatty acids of standard composition. Notably, the second substrate, rather than acyl-CoA, is acetyl-CoA. Consequently, the activity of this enzyme exclusively resulted in the formation of *sn*-1,2-diacyl-3-acetylglycerols or optically active asymmetric acetates of diacylglycerols [55,58]. Thus, the *sn*-1 and *sn*-3 positions of the glycerol residue are not always equivalent in the biosynthesis of TAGs.

Indeed, native *sn*-1,2-diacylglycerols, which were detected in minor amounts in the fatty oil of mature seeds of *Lunaria rediviva*, contained approximately 33% C20:1–C24:1 fatty acids. Given the observation that all of these acids acylated the *sn*-1,3 positions of the TAGs, it can be deduced that the aforementioned acids in the composition of native *sn*-1,2-DAGs are exclusively in the *sn*-1 position. Furthermore, the proportions of erucic and nervonic acids in native *sn*-1,2-DAGs were found to be 43 and 53 percent of their content in TAGs, respectively. In contrast, the proportion of gondoic acid in the *sn*-1 position was 71%, indicating the predominant acylation of the *sn*-1 position with 20:1 acyl and a low probability of the formation of *sn*-1,3-digondonoyl-2-acylglycerols. This assertion is well corroborated by our experimental data.

Concurrently, the demonstration of a stereospecific distribution of fatty acid acyls within the TAG structure necessitates a series of experiments that are methodologically intricate to validate the hypothesis. Nevertheless, precedents of stereoselectivity or non-equivalence of *sn*-1/3 positions in the biosynthesis of glycerolipids, including TAGs, are described in the literature. For instance, David Taylor’s research utilised patrial chemical deacylation, followed by the isolation of *sn*-2-, *sn*-1-, and *sn*-3-monoacylglycerols employing chiral HPLC, to examine the stereospecific distribution of C20:1–C24:1 fatty acids in diverse genotypes of high-erucic varieties of rapeseed (*B. napus*) [50]. The study revealed that, with a comparable amount of erucic acid in the TAGs, the distribution between the *sn*-1/3 positions could be either symmetrical or asymmetrical with the preferential acylation of the *sn*-3 position. To illustrate this, examine the Mercury genotype, which exhibited a 51.7% of 22:1 acid content in TAGs. Its content in the *sn*-1 and *sn*-3 positions was 58.0% and 59.6%, respectively, indicating equal proportions in these locations. Conversely, in the case of rapeseed of the Reston genotype, with a 22:1 acid content in TAGs of 29.5%, the distribution was found to be unequal between the *sn*-1 and *sn*-3 positions. In the *sn*-1 position, the content was found to be only 8.2%, whereas in the *sn*-3 position, its share was 42.8% of the total fatty acids. Conversely, the 20:1 acids exhibited a distinct distribution pattern, predominantly acylating the *sn*-1 position of the TAGs [50].

It is therefore logical to hypothesise that for the formation of symmetrical *sn*-1,3-dierucoyl-2-acyl-glycerols, the presence of two isoforms is necessary. The first of these is LPAAT, which has a specificity for erucoyl-CoA, and the second one is DGAT, which has a specificity for erucoyl-CoA on one hand and for *sn*-1-eucoryl-2-acyl-glycerol on the other. In the absence of at least one of these enzymes, the predominant formation of triacyl *rac*-1-erucoyl-2-acyl-3-acylglycerols becomes evident. This finding indicates that the presence of fatty acid elongases alone is insufficient to yield oils containing these acids. The appearance of a complex of enzymes with pronounced substrate specificity is necessary [59].

## 5. Conclusions

Isoforms of enzymes involved in the biosynthesis pathway of TAGs and phospholipids have been actively studied in recent years, since understanding their function and structure opens up ways to obtain transgenic oil crops producing vegetable oils with a specific structure of tracylglycerols, which may have not only technical but also nutraceutical value. In the context of biotechnology and genetic engineering of such producer plants, it is essential to detect and study plants that can serve as a source of genes encoding enzymes of fatty oil biosynthesis with prominent substrate specificity. In our study, we utilised a combination of elementary analyses and relatively simplified mathematical models, employing the example of studying TAGs in *Lunaria rediviva* seeds. This approach enabled us to make reasonably accurate assumptions about the presence of several enzyme isoforms in this plant species, and to propose an effective strategy for their search, based on the structure of the synthesized glycerolipids. The rationale underpinning this assertion is that the presence of a metabolite is indicative of the enzyme existence (or its isoform) responsible for its biosynthesis and, consequently, a gene encoding this enzyme. In our future research, we aim to perform a detailed study of the kinetics of fatty oil accumulation in ripening seeds of perennial honesty. We will also examine the features of biosynthesis and the accumulation of various molecular types of TAGs containing monounsaturated fatty acids with a very long chain. This will facilitate the identification of the stages of ripening at which biosynthesis of these target molecular types is most active and, consequently, the determination of the stages at which the number of transcripts of target genes and the activity of the enzymes encoded by them are maximal. This information will assist in the expeditious identification of these genes for their subsequent utilisation in the biotechnology of transgenic oil crops.

## Figures and Tables

**Table 1:** Fatty acid composition of TAGs from *Lunaria rediviva* seeds for the different sample preparation methods.

Fatty Acids, Mass%	Derivatisation Method (*n* = 3)		
	**Saponification**	**1%** $H_{2}{\mathrm{SO}}_{4}$/MeOH	**0.2 M TMSH**
16:0	*** 1.5±0.1	4.1±0.3	*** 1.4±0.1
18:0	* 0.1±0.0	0.2±0.0	* 0.1±0.0
18:1Δ9	** 18.6±0.1	16.0±0.6	** 18.4±0.4
18:2Δ9,12	12.2±0.2	12.1±0.1	12.3±0.3
18:3Δ9,12,15	** 1.5±0.1	2.1±0.1	** 1.6±0.1
20:1Δ11	** 9.9±0.0	13.0±0.5	** 9.7±0.1
22:1Δ13	48.0±0.3	47.4±1.2	48.2±0.7
24:1Δ15	*** 6.9±0.0	4.2±0.2	*** 6.9±0.1
Others FAs ^1^	1.4±0.1	0.9±0.1	1.4±0.1

^1^ Also contained 14:0, 16:1Δ7, 16:1Δ9, 18:1Δ11, 20:1Δ13, 20:2Δ11.14, 20:0, 22:0, 22:1Δ15, 24:0, 24:1Δ17 in amounts of 0.01–0.4%. Results of ANOVA test compared with 1% $H_{2}SO_{4}$ in methanol: *** -*p* < 0.001, ** -*p* < 0.01, * -*p* < 0.05.

**Table 2:** Characteristic fragment masses of DMOX derivatives of unsaturated fatty acids found in triacylglycerols of *Lunaria rediviva*.

Chain Length, C Atoms	M+, *m/z*	Ion Pairs with a 12 Amu Difference, *m/z*	-HC=CH- Bond Position	Fatty Acid
Monoenoic fatty acids				
16	307	168–180	-C_7_=C_8_-	16:1Δ7
16	307	196–208	-C_9_=C_10_-	16:1Δ9
18	335	224–236	-C_11_=C_12_-	18:1Δ11
20	363	252–264	-C_13_=C_14_-	20:1Δ13
22	391	280–292	-C_15_=C_16_-	22:1Δ15
24	419	308–320	-C_17_=C_18_-	22:1Δ17
Deinoic fatty acids				
16	305	168–180	-C_7_=C_8_-	16:2Δ7,10
		222–234	-C_9_=C_10_-	
18	333	196–208	-C_9_=C_10_-	18:2Δ9,12
		236–248	-C_12_=C_13_-	
20	361	224–236	-C_11_=C_12_-	20:2Δ11,14
		264–276	-C_14_=C_15_-	
22	389	252–264	-C_13_=C_14_-	22:2Δ13,16
		292–304	-C_16_=C_17_-	
24	417	280–292	-C_15_=C_16_-	24:2Δ15,18
		320–332	-C_18_=C_19_-	
Trienoic fatty acids				
16	303	168–180	-C_7_=C_8_-	16:3Δ7,10,13
		208–220	-C_10_=C_11_-	
		248–260	-C_13_=C_14_-	
18	331	196–208	-C_9_=C_10_-	18:3Δ9,12,15
		236–248	-C_12_=C_13_-	
		276–288	-C_15_=C_16_-	

FAs with the same ethylene bond positions but different chain lengths were not included in the table. The complete set of FAs and isomers of -C=C- bond positions is shown in Figure 1.

**Table 3:** Fatty acid compositions of *sn*-1,2,3, *sn*-2, and *sn*-1,3 positions of different neutral acylglycerols of mature *Lunaria rediviva* seeds; mass % (*n* = 9).

*sn*-Position	Lipid	Fatty Acids, Mass%								
		**16:0**	**18:0**	**18:1** Δ **9**	**18:2** Δ **9,12**	**18:3** Δ **9,12,15**	**20:1** Δ **11**	**22:1** Δ **13**	**24:1** Δ **15**	**Others**
Experimental composition										
*sn*-1,2,3	TAG	1.7±0.1	0.1±0.0	20.5±0.5	13.8±0.3	1.8±0.1	9.8±0.12	44.8±0.8	6.0±0.1	1.3±0.1
*sn*-1,2/2,3	*rac*-1,2-DAG	2.8±0.8	0.4±0.4	32.8±0.6	23.8±1.1	3.6±0.2	7.3±0.28	25.7±1.8	3.2±0.3	0.5±0.2
*sn*-2	*sn*-2-MAG	0.6±0.5	0.0±0.0	51.2±3.3	41.5±2.6	6.7±0.8	0.0±0.00	0.0±0.0	0.0±0.0	0.0±0.0
*sn*-1,2	*native*-DAG	7.5±0.8	0.9±0.3	20.7±0.5	36.3±0.9	3.8±0.3	7.0±0.72	19.4±1.6	3.2±0.4	1.2±0.1
Calculated composition										
*sn*-1,3	From DAG ^1^	0.4±0.5	0.0±0.0	0.2±0.5	0.0±0.0	0.0±0.0	13.1±0.7	73.1±1.4	10.2±0.4	3.1±0.4
	From MAG ^2^	2.2±0.3	0.1±0.0	5.1±2.1	0.7±0.9	0.0±0.0	14.5±0.2	66.4±1.3	8.8±0.3	2.1±0.1
*sn*-2 ^3^		4.3±2.1	1.0±1.1	49.6±2.2	38.3±2.7	6.4±0.4	0.2±0.3	0.0±0.0	0.0±0.0	0.2±0.3

^1^ Calculated from *rac*-1,2-DAGs by Formula (3). ^2^ Calculated from *sn*-2-MAGs by Formula (2). ^3^ Calculated from *sn*-1,2,3-TAGs and *rac*-1,2-DAGs by Formula (1).

**Table 4:** Enrichment factors (EFs) and selectivity factors (SFs) of incorporation of FAs into the *sn*-2 position of TAGs of mature seeds.

Factor	Fatty Acids, Mass%							
	**16:0**	**18:0**	**18:1**Δ**9**	**18:2**Δ**9,12**	**18:3**Δ**9,12,15**	**20:1**Δ**11**	**22:1**Δ**13**	**24:1**Δ**15**
EF ^1^	0.4±0.3	0.0±0.0	2.8±0.2	3.4±0.3	4.2±0.7	0.0±0.0	0.0±0.0	0.0±0.0
SF ^2^	0.4±0.3	0.0±0.0	2.8±0.2	3.3±0.3	4.1±0.7	0.0±0.0	0.0±0.0	0.0±0.0

^1^ Enrichment factor calculated by Formula (4). ^2^ Selectivity factor calculated by Formula (5).

## Data Availability

The original contributions presented in this study are included in the article. Further inquiries can be directed to the corresponding author.

## Supplementary Materials

The following supporting information can be downloaded at: https://www.mdpi.com/article/10.3390/plants14040612/s1, Figure S1: Flowering plant *Lunaria rediviva*, July 2023, territory of the Main Botanical Garden of the Russian Academy of Sciences; Figure S2: Seeds of ripe fruits of *Lunaria rediviva*, October 2023. Collected on the territory of the Main Botanical Garden of the Russian Academy of Sciences.

## Abbreviations

The following abbreviations are used in this manuscript:

ACC	Acetyl-CoA carboxylase
ACP	Acyl-carrying protein
DAG	Diacylglycerol
DGAT	Diacylglycerol acyltransferase
DMOX	4,4-dimethyloxazoline derivative of fatty acids
ECN	Equivalent carbon number
EF	Enrichment factor
FA	Fatty acid
FAME	Fatty acid methyl ester
FAS	Fatty acid synthase
FAX	Fatty acid export
GC	Gas chromatography
GPAT	*sn*-glycerol-3-phosphate-acyltransferase
HPLC	High-performance liquid chromatography
LPAAT	Lysophosphatidic acid acyltransferase
MAG	Monoacylglycerol
SF	Selectivity factor
TAG	Triacylglycerol
TLC	Thin-layer chromatography
TMSH	Trimethylsulfoniumhydroxid
VLCFAs	Very long-chain fatty acids
