# Supplementary material for: The Structure of Storage Triacylglycerols of Mature Seeds of Lunaria rediviva L., a Hyperaccumulator of Very Long-Chain Monounsaturated Fatty Acids, from the Perspective of Statistical Distribution Theories and New Insights Based on Simple Calculations
Source: Plants (Basel). 2025 Feb 18;14(4):612. doi: 10.3390/plants14040612 (PMC11859942; doi:10.3390/plants14040612)
Supplement: Supplementary file 1 [file plants-14-00612-s001.zip › plants-3478107-supplementary.pdf]

Supplementary files

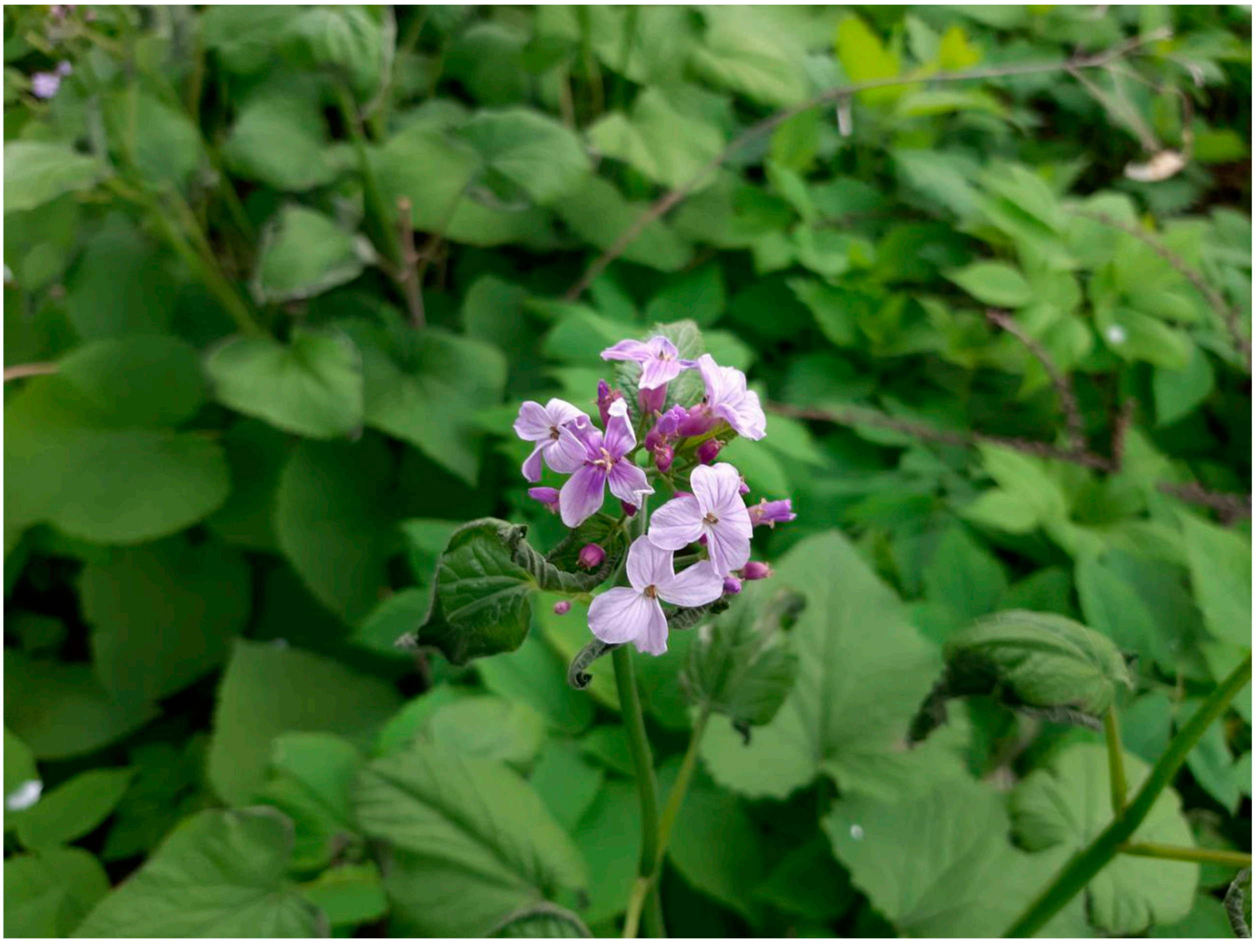

Figure S1: Flowering plant *Lunaria rediviva*, July 2023, territory of the Main Botanical Garden of the Russian Academy of Sciences

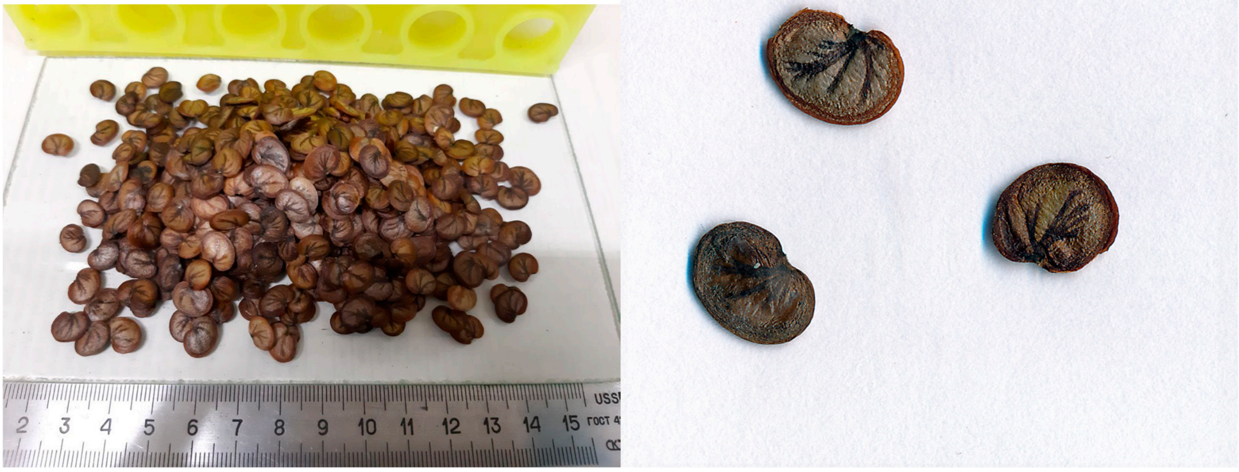

Figure S2: Seeds of ripe fruits of *Lunaria rediviva*, October 2023. Collected on the territory of the Main Botanical Garden of the Russian Academy of Sciences
